# Child Bilingual Acquisition of Spanish Dative (Non-)clitic Doubling Structures: A Case Study Approach to Home and Community Input Conditions

**DOI:** 10.1007/s10936-026-10284-3

**Published:** 2026-06-27

**Authors:** Raquel Fernández Fuertes, Silvia Sánchez Calderón

**Affiliations:** 1 Departamento de Estudios Ingleses, Facultad de Filosofía y Letras, University of Valladolid Language Acquisition Lab, Plaza del Campus Universitario s/n, 47011 Valladolid, Spain; 2 Departamento de Filologías Extranjeras y sus Lingüísticas, Facultad de Filología, National University of Distance Education, Paseo Senda del Rey 7, 28040 Madrid, Spain

**Keywords:** Dative (non-)clitic doubling structures, Emergence, Incidence, Child-directed speech, L1 bilingualism, HL bilingualism

## Abstract

We investigate the acquisition of dative clitic doubling (CD) and non-clitic doubling (NCD) in Spanish–English child bilinguals who are exposed to Spanish as their first language (L1) (a) in a minority language context (i.e., H(eritage) L(anguage) bilingual children) and (b) in a majority language context (i.e., L1 bilingual children) to address how input and social conditions shape the early bilingual language acquisition stages. These data are compared to those of Spanish monolinguals. The two constructions emerge at around the age of 2 in the two bilingual child groups and in the monolingual group. This points to the fact that the three child groups have equally acquired the shared complex predicate structure upon which dative CD and NCD structures are built. Moreover, dative NCD constructions are less frequent than dative CD in the two bilingual groups and in the monolingual group, which is in tune with the child-directed speech production patterns. Thus, taking the Spanish monolinguals’ results as the baseline, the social and/or the family context in which Spanish is being acquired from early on as an HL or as an L1 does not seem to interfere in the acquisition of the grammatical properties and patterns of use of these constructions.

## Introduction

This study investigates the interplay between family and community input^1^ in early bilingual first language acquisition of Spanish grammar with a particular focus on child bilingual acquisition of two types of morphosyntactic variants, namely, dative clitic doubling (CD) and non-clitic doubling (NCD) structures in Spanish. Two input conditions have been considered in this analysis, one in which the children are exposed to Spanish as their first language (L1) in a minority language context (i.e., H(eritage) L(anguage) bilingual children) and children that are exposed to L1 Spanish in a majority language context (i.e., L1 bilingual children). That is, a comparison is established between (i) children who are exposed to Spanish both in their family setting as well as in the community (L1 bilingual children) and children who are exposed to Spanish only in their family setting (HL bilingual children); and (ii) L1 and HL bilingual children and the child-directed speech they are exposed to. The constructions that are explored involve two types of dative alternation structures: the ones in which a dative CD structure (1a) alternates with a ditransitive verb as a dative NCD construction headed by the preposition ‘*a*’ (that is, to in English) (1b), and the ones in which a dative CD structure (2a) alternates with a monotransitive verb as a dative NCD construction with the preposition ‘*para*’ (that is, for in English) (2b). The syntactic properties that underlie the structures at stake are discussed in Sect. “Main definitory properties and idiosyncrasies of early child bilingualism based on the role played by home and societal language exposure”.

**Table 1:** Spanish–English bilingual corpora selected

Language group	Corpora	Child	Age range (selected)	Age range (corpus)	Social context	Home context
HL bilinguals	Deuchar	Manuela	1;03–3;03	1;03–3;03	English (UK) in crèche and the street	Mostly Spanish by both parents (father’s L1 and mother’s L2) Also English to a lesser extent by grandmother (one day a week)
	Perez	Carla		2;00–3;03	English (USA) in the street and preschool from 3;00 to 3;03 Spanish to a lesser extent in playgroup from 2;00–3;03 and visits to Spanish-speaking countries between 4 and 6 weeks a year	Mostly Spanish by both parents (mother’s L1 and father’s L2) and babysitter from 1;11 to 2;11 Also English by babysitter from 1;03–1;11
L1 bilinguals	FerFuLice	Leo	1;01–3;01	1;01–6;11	Spanish (Spain) in the street and day care for 3 h a day on weekdays Also English to a lesser extent in the summer when visiting the USA	Spanish by father (L1): one parent-one language Also English to a lesser extent in occasional visits to the USA for two months every summer
		Simon		1;01–6;11	Spanish (Spain) in the street and day care for 3 h a day on weekdays Also English to a lesser extent in the summer when visiting the USA	Spanish by father (L1): one parent-one language Also English to a lesser extent in occasional visits to the USA for two months every summer

**Table:** 

(1) Dative (non-)clitic doubling ditransitive structures (ENSEÑAR-‘SHOW’)			
a	Enséñale	la foto a la mamá	[dative CD]
	teach.2p.sg.imp.him.cl.dat.3p.sg	the picture to the mummy	
	‘Show the mummy the picture’		
b	Enseña	la foto a la mamá	[dative NCD with ‘*a*’]
	teach.2p.sg.imp	the picture to the mummy	
	‘Show the picture to the mummy’		

**Table:** 

(2) Dative (non-)clitic doubling monotransitive structures (COMPRAR-‘BUY’)					
a	Le	compraré	un	juguete a mi sobrino	[dative CD]
	him.cl.dat.3p.sg	buy.1p.sg.fut	a	toy to my nephew	
	‘I will buy my nephew a toy’				
b	Compraré	un	juguete para mi sobrino	[dative NCD with ‘*para*’]	
	buy.1p.sg.fut	a	toy for my nephew		
	‘I will buy a toy for my nephew’				

Animacy plays a crucial role in determining the distribution of the phenomenon of CD in Spanish, namely, the indirect object (IO) is required to exhibit animate or human features so as to hold clitic and non-clitic doubling. However, when the direct object (DO) is animate, direct object marking blocks this alternation and this is especially the case in the formation of CD (3a), where the DO and the IO are marked with the preposition ‘*a*’ (to) and thus, the disambiguation of two constituents preceded by this preposition is a complex cue to acquire by children.

**Table:** 

(3) a	¿?/*El maestro	presentó	a	su	mujer	a	los	alumnos		
	The teacher	present.3p.past	to	his	wife	to	the	students		
	‘The teacher presented his wife to the students’									
b	El	maestro	les	presentó	a	su	mujer	a	los	alumnos
	The	teacher	them.cl.dat.3p.sg	present.3p.past	to	his	wife	to	the	students
	‘The teacher presented his wife to the students’									
[Fischer & Navarro, 2016, p. 46]										

Taking as a point of departure the two dimensions of input source referred to before (i.e., home versus community input) and input received from child-directed speech, we investigate the emergence and the incidence patterns of the two types of Spanish dative complex predicates when Spanish–English simultaneous bilingual children are exposed to Spanish from early on as an HL in the home setting or as an L1 both at home and as the language of the community. We also analyze the exposure to dative CD and NCD constructions that the two bilingual child groups receive in their respective child-directed speech. A comparative analysis of the results examined in the HL and the L1 bilingual children, as well as the adult input child-output production patterns, will be carried out with Spanish monolingual children.

This paper is organized in 6 sections. Sect. “Main definitory properties and idiosyncrasies of early child bilingualism based on the role played by home and societal language exposure” addresses the grammatical properties underlying dative CD and NCD constructions in Spanish. Sect. “Previous bilingual and monolingual studies on the emergence and adult input effects of dative (non-)clitic doubling structures in Spanish” explores the properties and idiosyncrasies of early child bilingualism, with particular attention to the role of the home context and the wider social environment. Sect. “The empirical study” reviews previous empirical works on the acquisition of the constructions under scrutiny. Sect. “Conclusions” formulates the research questions (RQs) guiding the study (Sect. 5.1) and outlines the methodology employed, including an overview of the participants (Sect. 5.1.1), the procedures of data selection and classification (Sect. 5.1.2), and the criteria for excluding instances from our data analyses (Sect. 5.1.3). Section 5.2 reports the results, which are subsequently discussed in Sect. 5.3 in relation to findings from prior research. Finally, Sect. 6 offers concluding remarks and outlines directions for future investigation.

## Grammatical Properties of Dative (Non-)clitic Doubling Structures in Spanish

Spanish dative CD and NCD structures are characterized by the overt or the null realization of a dative clitic pronoun in the third person form, namely, ‘*le*’ (singular, masculine or feminine; him/her) or ‘*les*’ (plural, masculine or feminine; them) (e.g., Cuervo, 2003; Demonte, 1995). In particular, these dative clitics are overtly realized in pre-verbal position in the formation of dative CD (4a) and co-appear with an animated and specific nominal IO^2^ headed by the prepositions ‘*a*’ (to) or ‘*para*’ (for) with which they share dative Case and recipient or beneficiary theta roles as well as number and gender features. Alternatively, dative clitics can also be non-overtly realized in their alternating prepositional counterparts (4b), namely, dative NCD. Semantic differences have been argued to occur between the two constructions: while CD entails a change in the state of the IO, the NCD variant simply describes the activity and does not specify whether a change of state has occurred (Gómez Seibane, 2023).

**Table:** 

(4) a	Enséña**le**_i_	la foto [a la mamá]_i_	[dative CD]
	teach.2p.sg.imp.him.cl.dat.3p.sg	the picture to the mummy	
	‘Show the mummy the picture’		
b	Enseña	la foto a la mamá	[dative NCD with ‘*a*’]
	teach.2p.sg.imp	the picture to the mummy	
	‘Show the picture to the mummy’		

Unlike the structures in (4), for which the verbal head is ditransitive since it selects a DO and an IO, the verb in dative NCD with ‘*para*’ (5b) and their dative CD counterpart (5a) are monotransitive predicates that subcategorize for a DO, along with a prepositional adjunct. NCD with ‘*para*’ do not allow the alternation with dative CD headed by the preposition ‘*para*’ (5c).

**Table:** 

(5) a	**Le**_i_	compraré	un	juguete [a mi sobrino]_i_	[dative CD]
	him.cl.dat.3p.sg	buy.1p.sg.fut	a	toy to my nephew	
‘I will buy my nephew a toy’					
b	Compraré	un	juguete para mi sobrino	[dative NCD with ‘*para*’]	
	buy.1p.sg.fut	a	toy for my nephew		
	‘I will buy a toy for my nephew’				
c	*Le_i_	compraré	un	juguete [para mi sobrino]_i_	[dative CD]
	him.cl.dat.3p.sg	buy.1p.sg.fut	a	toy for my nephew	
‘I will buy my nephew a toy’					

Considering the properties that underlie dative CD and NCD in Spanish, there is a debate in the literature as to whether the two structures syntactically derive from one another (e.g., Demonte, 1995) or, on the contrary, they do not exhibit a derivational relationship. Following the derivational approach, dative CD predicates have been argued to be derivationally associated with their dative NCD counterparts (e.g., Demonte, 1995). With regards to the non-derivational approach, two standpoints appear: one in which dative CD and NCD in Spanish are syntactically and semantically different (Cuervo, 2003), and another one in which the two constructions exhibit a common complex predicate or small clause (SC) structure, as has been proposed for English (Snyder, 2001). By contrast and, in line with Snyder’s (2001) Complex Predicate Parameter,^3^the two Spanish complex dative predicates analyzed in this study are argued to form a natural syntactic class of complex predicate constructions with a shared parametric property. Complex dative predicates are also present in other languages such as English, the other first language (L1) of the bilingual children in our study. These structures are characterized by the overt realization of the prepositions to and for in the construction of to-datives (6a) and for-datives (6c), and their absence in the formation of double object constructions (6b and 6d) (e.g., Snyder & Stromswold, 1997).

**Table:** 

(6) a. Lisa told an interesting story to her friend	[to-dative]
b. Lisa told her friend an interesting story	[double object construction]
c. Our parents made a nice cake for Peter	[for-dative]
d. Our parents made Peter a nice cake	[double object construction]

This study takes as a starting point the study conducted by Sánchez Calderón and Fernández Fuertes (2020) and focuses on whether Spanish CD and NCD predicates show differences in the timing of acquisition and in their production patterns as a result of the differences in the amount of exposure to two input sources in Spanish from birth, namely, in the family setting (in the HL bilinguals) and simultaneous exposure to this language at home and in the community (in the L1 bilinguals). We particularly explore the ages of first occurrence and the incidence patterns in the child output and establish comparisons of these findings with the production patterns in their corresponding child-directed speech. Given the results reported by previous studies on the input effects on language acquisition (e.g., Fernández Fuertes and Liceras, 2018; Yang, 2016), a higher exposure to Spanish from an early stage, as in the case of L1 bilinguals, is expected to exhibit an earlier emergence and higher frequency rates in the use of CD and NCD when compared to HL bilinguals whose exposure to the language is restricted to the home context.

## Main Definitory Properties and Idiosyncrasies of Early Child Bilingualism Based on the Role Played by Home and Societal Language Exposure

Bilingual first language acquisition involves the exposure to two languages from birth. Work on this type of acquisition context has systematically shown that language dominance, a definitory and salient property of bilingual acquisition, is in fact related to issues such as the relative amount of input that bilinguals receive (Montrul, 2016; Pires & Rothman, 2009). In this respect, a difference in input has been argued to be at the core of the difference between two bilingual first language acquisition contexts: the acquisition of two balanced first languages (L1 bilingualism) and the acquisition of a first language (that is, their societal language) and a heritage language (HL bilingualism). In the first case, a certain balance exists between the two L1s, and this entails that the two languages exhibit a somehow equal amount of input and that, in some cases, both languages are present in one way or another in the home context and in the child’s social context (e.g., Fernández Fuertes & Liceras, 2018). In the second case, the child’s exposure to the HL is restricted to the home context, and their societal language is present in the community (e.g., Hurtado & Montrul, 2021; Montrul, 2016; Pires & Rothman, 2009). Nonetheless, individual difference factors are reflected in this respect since, while some children are chiefly exposed to the HL at home by both parents and their other L1 is present in society, others are exposed to a mixture of the HL and the L1 both at home and in the community (Paradis, 2023). In any case, access to the HL is more constrained with respect to L1 bilingual children. At the early acquisition stages, HL bilingual children are prone to exhibit different acquisition paths in the HL when compared to children that are primarily acquiring this language in the community. These acquisition differences between HL bilinguals and monolinguals (almost) disappear after years of schooling in the societal language, making them comparable to the acquisition patterns of monolingual children. However, not all HL speakers experience bilingual first language acquisition, and some are exposed to the societal language or the HL as a second language (L2) from an early stage as a result of the migration of first, second or third generations (Paradis, 2023). This difference in the type and amount of input in the bilingual’s two languages has been said to trigger different acquisition paths and patterns when bilinguals’ L1(s) and HL are compared to their monolingual peers or even when different bilinguals are compared (Montrul, 2016; Montrul et al., 2015). In the case of HL Spanish, previous studies have pointed out that exposure to the HL is mainly achieved through auditory input in social interactions in the family context (Montrul & Potowski, 2007; Pires & Rothman, 2009). Furthermore, many HL speakers do not receive (or receive limited) schooling in the HL (Montrul, 2016). In relation to quantity of input and both in the case of L1 and HL bilingualism, the amount of exposure to a particular structure in the child-directed speech has proven to have positive effects in the bilingual and monolingual children’s output (e.g., Yang, 2016). This has been reported in different linguistic domains, namely, lexicon (e.g., Genesee, 2002), phonology (e.g., Polka & Sundara, 2003) and morpho-syntax (e.g., Fernández Fuertes and Liceras, 2020). However, to the best of our knowledge, earlier research on bilingual child language acquisition, in general, has not considered in the same studies an approach that examines not only input frequency effects, as measured in the child-directed-speech children are exposed to, but also the role played by the status of languages the children have been surrounded by since birth, namely, as a societal language versus as an HL with the corresponding amount of input differences this status involves. Comparing bilinguals for whom a specific language (Spanish, in the case of the present study) has a different status (as a majority language versus as a minority language) allows us to put in the spotlight issues such as the potential differences between L1 and HL bilingual contexts, as well as to compare these two contexts in terms of the nature of the parental input. This contributes to the characterization of input and how it shapes the acquisition processes. Very few studies have compared the developmental acquisition patterns followed by HL and L1 bilingual children, and how these relate to their monolingual counterparts, in general, or in the specific case of Spanish complex dative predicates. This is the gap this study intends to help fill in.

## Previous Bilingual and Monolingual Studies on the Emergence and Adult Input Effects of Dative (Non-)clitic Doubling Structures in Spanish

Earlier studies on the acquisition of Spanish dative CD and NCD constructions have examined, among others, the following issues: (a) the age of onset of these two predicates in child spontaneous production data (e.g., Sánchez Calderón & Fernández Fuertes, 2020 in Spanish–English bilinguals; Torrens & Wexler, 2000 in Spanish monolinguals); and (b) the use of the two target constructions in the language of HL bilingual adults, as examined through oral production data (e.g., Silva Corvalán, 1997) and acceptability judgment data (e.g., Montrul, 2010). For one of the two constructions, namely, dative CD, prior research has additionally relied on oral production data (e.g., Irizarri, 2014) and structural priming experiments (Hurtado & Montrul, 2021). Child-directed speech and child output have also been explored in Spanish–English bilingual spontaneous production data (e.g., Sánchez Calderón & Fernández Fuertes, 2020). These issues are described below as they are cardinal for the study that we present in Sect. “Conclusions”. Very few studies have examined the emergence of Spanish dative CD and NCD in Spanish–English bilingual child data (e.g., Sánchez Calderón & Fernández Fuertes, 2020)^4^ and Spanish monolingual child data (e.g., Torrens & Wexler, 2000). The data analysis of the 9 Spanish–English bilingual children in Sánchez Calderón and Fernández Fuertes (2020) reveals that Spanish dative CD and NCD emerge at around the age of 2 (*t*(5) = -2.029, *p* = .135), as examined in child spontaneous production available in the CHIld Language Data Exchange System (CHILDES; MacWhinney, 2000). These statistical results have been argued to lie in the fact that Spanish–English bilinguals are building a common, and thus, syntactically underived, complex predicate or SC construction (Snyder & Stromswold, 1997). Nonetheless, the emergence of NCD seems to be delayed as a result of the mediating dative Case and goal or beneficiary theta role assignment of the prepositions in these structures.^5^ In the case of Spanish monolingual child spontaneous production data, Torrens and Wexler (2000) analyze the age of first occurrence of Spanish dative CD utterances in a case study (María; the Ornat corpus, CHILDES). The children’s ages range from 1;07 to 3;11.^6^ The results showed that dative CD appears between 1;07 and 2;03. The use of dative CD and NCD in Spanish has been extensively explored in adult HL contexts (Montrul, 2010 and Silva Corvalán, 1997 in the production of the two target structures; Irizarri, 2014 and Hurtado & Montrul, 2021 in CD). Silva Corvalán (1997) examines the oral production of second-generation bilingual adults in Los Angeles and observes that HL Spanish speakers have a preference for using dative NCD over CD. Contrasting results are reported by Montrul (2010) who conducts a comparative study with HL Spanish adults and L2 Spanish learners on their knowledge of dative CD and NCD via a written acceptability judgment task. The results of this study reveal that the HL speakers show higher acceptance rates for the use of dative CD when compared to dative NCD. As for elicited oral production, the HL speakers produce more dative clitics overall than the L2 speakers. However, for this oral task, the HL speakers reflect lower rates of dative clitics than Spanish-dominant speakers or possibly monolingual Spanish speakers. Thus, it seems that, at least in the case of adults, the data elicitation technique could be affecting the different outcomes. In the context of HL Spanish in the Netherlands, Irizarri (2014) analyzes dative CD predicates by HL Spanish adults (N = 24; age range: 21–78) who are compared to Spanish monolingual adults from Chile (N = 16; age range: 20–35). Data are extracted via a corpus of interviews that elicited spontaneous speech. Results show statistically non-significant differences in the high production rates of dative clitics in dative CD between the HL Spanish adults and the Spanish monolingual speakers, as evidenced by a Mixed Effects Logistic Regression analysis. These results lead Hurtado and Montrul (2021) to examine whether structural priming facilitates the use of dative clitics in HL Spanish bilinguals at an American university (N = 24). A posttest was also conducted based on a dative clitic elicitation picture description task following a structural priming treatment. In their study, findings reflect that the HL Spanish speakers show more monolingual-like preferences for dative clitics at baseline than L2 speakers of Spanish. All the groups show boosts in dative clitic production due to priming; nevertheless, the HL and the L2 groups exhibit longer-lasting effects of priming than the monolingual groups. Thus, construction frequency is crucial in the production of dative CD. Along with the emergence of dative CD and NCD, adult input effects in the home setting and, in particular, the role played by child-directed speech in the children’s production of these two structures have also been explored. While this field has not been widely investigated in Spanish–English bilinguals (e.g., Fernández Fuertes & Sánchez Calderón, 2021), a substantive number of studies has explored child-directed speech effects in monolingual children’s use and ages of first of occurrence of the two constructions at stake in English, the bilinguals’ other L1 (e.g., Campbell & Tomasello, 2001). As for Spanish bilingual children, previous findings reported by Fernández Fuertes and Sánchez Calderón (2021) reveal that the higher production rates of dative CD over NCD in the child-directed speech is comparable to that in the Spanish–English bilingual children’s Spanish output, as extracted from CHILDES (MacWhinney, 2000). These data reflect similar outcomes to the ones examined in their Spanish monolingual peers. Although Spanish–English bilinguals have evidenced to pattern closer to their child-directed speech in the use of the two constructions, the actual role of child-directed speech effects has not been disentangled as to whether these effects are equally seen in HL and L1 bilinguals given the different context of exposure to the language they exhibit (that is, home-only versus home and community). In English, and in line with the Hypothesis of Ordered Input (Borer & Wexler, 1987), the results derived from Campbell and Tomasello (2001) show the same production preference for double object constructions over their prepositional alternating counterparts in the child-directed speech and in the monolingual children’s output (age range: 1;02–5;00, *p* > .01, binominal test). Snyder and Stromswold (1997) also observe analogous effects regarding the production of double object constructions (average: 73.2%) and to-datives (average: 26.6%) in the child-directed speech, as analyzed with the verb ‘give’. Nonetheless, the frequency with which these structures are used by the adults does not correlate with the English monolingual children’s emergence order pattern (double object constructions > to-datives; *p* > .10).

## The Empirical Study

### Research Questions

In light of the syntactic underpinnings (Sect. “Main definitory properties and idiosyncrasies of early child bilingualism based on the role played by home and societal language exposure”) and earlier empirical studies on the HL and L1 bilinguals’ acquisition (Sects. “Previous bilingual and monolingual studies on the emergence and adult input effects of dative (non-)clitic doubling structures in Spanish” and “The empirical study”) of dative CD and NCD in Spanish, two RQs have been formulated:

#### RQ 1

Does the source of input (namely, home setting versus community environment) that bilingual children have been exposed to from birth trigger differences (or lack thereof) between HL bilingual children and L1 bilingual children with regards to the acquisition of the syntactic status that relates Spanish CD and NCD, as analyzed in the emergence and the incidence patterns of these structures? And are the acquisition patterns of these two constructions similar or different from the ones followed by Spanish monolingual children?

Given the debate that has arisen in the literature as to whether HL bilinguals pattern closer to L1 bilinguals (Pires & Rothman, 2009), or whether they reflect patterns that are closer to L2 speakers (Montrul et al., 2015), we set to investigate the initial stages of Spanish bilingual acquisition in order to elucidate whether the type and amount of Spanish input source that the two bilingual child groups have been exposed to is optimal for the acquisition of Spanish CD and NCD (Montrul, 2016).^7^ Taking Sánchez Calderón and Fernández Fuertes’ (2020) study as a starting point, we focus on the analysis of the two dative (non-)clitic doubling predicates in Spanish to address the issue of whether the reduced Spanish home input received by the HL bilingual children will reflect delayed emergence patterns of the two constructions under scrutiny than the L1 bilinguals. Furthermore, and given that the two target groups involve bilingual children, a comparison with Spanish monolingual children will help complete the picture of the role that input quantity exhibits in the acquisition of dative CD and NCD. In this respect, a hierarchy could be established in terms of the amount of input children receive in Spanish, as in (7).

**Table:** 

(7) Continuum of amount of input in Spanish			
	Monolingual children	L1 bilingual children	HL bilingual children
Home context	+ + +	+ (by one parent, and shared with English, the other L1)	+ + (90% by both parents + 10% babysitter from 1;11 to 2;11, and shared with English by babysitter from 1;03–1;11)
Social context	+ + +	+ + +	−

The monolinguals are in one extreme of the continuum since their full input is in Spanish in the home and in the social context. While the HL bilinguals are in the other extreme of the continuum, as their exposure to Spanish is limited to the family context by both parents (+ +), the L1 bilinguals are exposed to Spanish both in the home setting chiefly by their father ( +) and in the social context. By analyzing the child production across the two bilingual groups and further comparing it to that of the monolingual group, we aim at determining whether the similar patterns that have been shown to appear in the Spanish–English bilingual children’s emergence of dative CD and NCD (Fernández Fuertes & Sánchez Calderón, 2021) are kept constant regardless of the amount of input bilingual children receive from their parents and other caregivers. Thereby, RQ 1 aims to shed light on whether there is a direct correlation between family and/or community input quantity in the bilinguals’ acquisition of the syntactic patterns that relate dative CD and NCD predicates in Spanish. More specifically, if the source of input determines the acquisition process, a difference should be seen across the three participant groups regarding the age of onset and developmental incidence patterns of the two constructions at stake. This divergence will be particularly reflected when comparing across the two Spanish–English bilingual child groups and the Spanish monolingual child group. In the case of the latter and, given the continuum of the source of input in Spanish both in the social context and in the home setting (Table 7), further differences between the L1 bilinguals and the HL bilinguals are predicted to be evidenced in the onset and in the incidence patterns of the two target structures. If, however, it is not only a matter of the input source (home versus society) but of meaningful and sufficient input,^8^ all three groups could behave in the same way or at least so in the two bilingual groups.

#### RQ 2

Is the relative frequency of exposure to Spanish dative CD and NCD that the HL bilingual children and the L1 bilingual children receive in the home context also reflected in the children’s incidence and onset patterns of the constructions under scrutiny? And are these child-directed speech and child output patterns in line with the ones examined in their corresponding Spanish monolingual peers?

The two bilingual child groups differ in the child-directed speech quantity they receive in Spanish in the home context. While the adult family input presents a double source, namely, Spanish and English, in the L1 bilingual children, the adult input is exclusively in Spanish for the HL bilingual children. Therefore, the HL bilingual children are more exposed to Spanish in the home setting when compared to the L1 bilingual children because, in the case of the HL bilinguals, the basic pattern of home language exposure is one language at home (namely, Spanish) in contrast to that in the L1 bilinguals that make use of the so-called one-parent one-language strategy. In particular, the L1 bilingual group involves a set of twins that are exposed to Spanish at home mainly from their father and to English, their other L1 and also their mother’s L1. Taking the results observed in earlier empirical works (Fernández Fuertes & Sánchez Calderón, 2021; Irizarri, 2014; Montrul, 2010, 2016), similar findings are to be expected in the HL bilingual children and in the monolingual children with regards to the incidence of the two constructions under analysis. These outcomes are predicted to be the case regardless of the amount of exposure to Spanish in the home context. In this respect, the HL bilingual children and their monolingual counterparts should both demonstrate different patterns of use and emergence for the two target predicates, as it was also reported for the L1 bilinguals. Namely, dative CD structures are expected to show a higher incidence and an earlier age of first occurrence than dative NCD, and this will be equally portrayed in the child output and in the child-directed speech (e.g., Yang, 2016). All in all, the predictions stated for RQ 2 account for optimal input as a crucial variable for the acquisition of the grammatical properties that underlie the two Spanish target predicates. This means that our predictions argue against those studies that advocate that home input matters in language development more than input from outside the home for pre-school age children (Gutiérrez-Clellen et al., 2008). Hence, if sufficient input, regardless of the source (home and/or community), is in line with the children’s output, even in the child group that receives more reduced input in Spanish (namely, the HL bilinguals) as it is restricted to the home context and, thus, exposure to this language is not present in society (Table 7b), similar developmental incidence and emergence patterns are expected to be reflected in the output of the two Spanish–English bilingual child groups as well as in that of their respective Spanish monolingual peers. That is, if the three child groups receive meaningful and optimal input in Spanish dative CD and NCD, differences should not be seen in the production and onset patterns of the two structures and, therefore, child-directed speech quantity could be argued to be sufficient for the acquisition of the syntactic underpinnings of both structures.

**Table:** 

(7b) Continuum of amount of input in Spanish (RQ2). Further details regarding the home and society input that the three child groups receive are provided in Table 1 (for HL and L1 bilinguals) and in Table 2 (for Spanish monolinguals)			
	Monolingual children	HL bilingual children	L1 bilingual children
home context	only Spanish by both parents	mostly Spanish by both parents (one language of exposure at home)	Spanish by one of the parents and other caregivers (one parent-one language strategy) and shared with the other L1
	+ + +	+ +	+
society context	only Spanish	mostly English (crèche and street)	mostly Spanish (crèche and street)
	+ + +	−	+ +

In line with the prediction referred to above and, following the Hypothesis of Ordered Input (Borer & Wexler, 1987), the three child groups are also expected to reflect similar findings in the use of the two (non-)clitic doubling predicates. In particular, if, in the adult grammar, one structure (i.e., dative CD) is more frequent in use than its alternating predicate counterpart (i.e., dative NCD), this could be reflected not only in the children’s output, but also in the children’s emergence patterns (Yang, 2016). This is expected to be the case in the Spanish monolingual children and the Spanish–English bilingual children alike and, regarding the latter, in both the L1 bilinguals and the HL bilinguals considering that the adult input is also optimal in both bilingual child groups for the acquisition of the structures under consideration (Montrul, 2016).

### The Heritage Language and the First Language Spanish Bilingual Children

To address the two RQs outlined above, we have selected 4 Spanish–English bilingual children from the CHILDES project (MacWhinney, 2000), a freely available database (childes.talkbank.org). For the selection of these children, we have considered two types of Spanish input exposure, namely, home input and social input. While, in the L1 bilingual children, the exposure to the language under analysis is shared with the other L1 at home via the one parent-one language strategy and it is also the language of the community, the HL bilingual children are only exposed to Spanish in the home context and the language of the social context for this bilingual child group is chiefly their other L1. As displayed in Table 1, out of the 4 Spanish–English bilinguals selected, 2 children are HL bilinguals (2 girls), and their ages range from 1;03 to 3;03; and 2 children are L1 bilinguals (2 boys) whose ages range from 1;01 to 3;01.^9^ Although the two bilingual child groups cover a wider age range in the corpora available in CHILDES, as also shown in Table 1, we have carried out a study in which the children’s ages are comparable for data analyses, that is, we focus on the early stages of acquisition (ages 1;03–3;03) for which child production is also more linguistically restrained.

Regarding the social background of the two bilingual child groups, Manuela (the Deuchar corpus) was born and raised in Brighton (England) and Carla (the Perez corpus) lived in the USA, namely, Michigan. In Simon and Leo, the set of twins in the FerFuLice corpus were born and raised in Salamanca (Spain). Therefore, the 4 bilingual children selected in this study lived in a monolingual social context, namely, the UK in Manuela, the USA in Carla and Spain in Simon and Leo. This means that, as shown in Table 1, Spanish was more present in the set of twins when considering the amount of input they received at home and in society (crèche and street) altogether, and it was less present in the two HL bilinguals. Although Manuela’s and Carla’s both parents addressed their children in Spanish (being the L1 in Manuela’s father and Carla’s mother and the L2 in Manuela’s mother and Carla’s father), Spanish was not present in Manuela’s community input (she was exposed to English in crèche and in the street) and not highly present in Carla’s community input (she was exposed to Spanish in a playgroup from 2;00 to 3;03 and occasional visits to Spanish-speaking countries between 4 and 6 weeks a year; and to English in the street as well as in preschool from 3;00 to 3;03). The L1 bilinguals were exposed to this language at home chiefly from one of their parents (namely, their father), based on Ronjat’s (1913) one-parent-one-language approach, as well as from other caregivers (for example, aunts or uncles, grandparents and investigators) for whom Spanish is also their L1. The set of twins were also exposed to Spanish in day care for 3 h a day on weekdays. Outside the home context, English was present to a lesser extent when visiting the USA every summer for two months. As for the HL bilingual children, the exposure to Spanish in the home context was mainly Spanish and it came from both parents. In the Deuchar corpus, Spanish was the L1 for her father and the L2 for her mother who learned it in adulthood (note that the metadata in CHILDES do not specify the mothers’ proficiency level in Spanish). In the Perez corpus, Carla was exposed to 90% Spanish from both parents, being the L1 for the mother and the L2 for the father at a proficient level, and to 10% Spanish (from 1;11 to 2;11) and English (from 1;03–1;11) from a babysitter. The adults’ interactions with the two bilingual child groups (that is, the child-directed speech) constitute the adult input examined in this study. As available in the longitudinal corpora recorded in CHILDES and, more specifically, the age range selected, we have primarily analyzed the input from both parents as well as other caregivers in all the children. In the case of the HL bilinguals, Manuela’s adult input involves her mum (an L2 Spanish speaker)^10^ and her father (an L1 Spanish speaker) and, with regards to Carla’s input, the child-directed speech comes from her mother (an L1 Spanish speaker) and her father (an L2 Spanish proficient speaker), along with the investigator (an L1 Spanish speaker). The set of twins (L1 bilinguals) received input from their father, their grandmother, and investigators (L1 Spanish speakers). The two Spanish monolingual children’s child-directed speech is mainly constituted by both parents and, in the case of Emilio, other adults, being Spanish monolingual speakers in all cases.^11^

The two bilingual child groups have been compared to a group of 2 Spanish monolingual children (1 girl and 1 boy), also available in CHILDES, and for whom the home input and the social input were exclusively in Spanish. As illustrated in Table 2, and similar to the data selected from the Spanish–English bilingual children (Table 1), the Spanish monolingual children have also been selected taking into consideration all the longitudinal corpora available in CHILDES.

**Table 2:** Spanish monolingual children selected

Language group	Corpora	Child	Age range selected	Corpus age range	Social context	Home context
Spanish monolinguals	LlinasOjea	Irene	0;11–3;02	0;11–3;02	Spanish	Spanish
	Vila	Emilio		0;11–4;08	Spanish	Spanish

In particular, we have analyzed 2 corpora (LlinasOjea and Vila) at the early language acquisition stages, namely, from 0;11 to 3;02.^12^ The onset in the Spanish monolingual children’s age range selected is earlier than in the two bilingual child groups since we consider the initial developmental language delay that bilingual children develop with respect to their respective monolingual peers (Meisel, 2004). Along with the child output, child-directed speech utterances have also been analyzed in the three child groups. With adult input-based analyses, we aim to examine the adult input potential effects to the Spanish exposure in the children’s use and emergence of the two Spanish (non-)clitic doubling predicates.

### Data Selection and Classification

Our data have been codified considering the type of (non-)clitic doubling structure, including the two types of prepositional dative NCD, that is, those ones headed with the prepositions ‘*a*’ (8) and ‘*para*’ (9), and dative CD (10). As for dative NCD with ‘*a*’, the periphrastic verb ‘*va a hacer*’ (i.e., she is going to do) selects the DO-determiner phrase (DP) ‘*otra*’ (i.e., another one) and the IO-prepositional phrase (PP) ‘*a mamá’* (i.e., to mummy). With regards to dative NCD with ‘*para*’, the verb ‘*va a comprar*’ (i.e., (he) is going to buy) selects the DO-DP ‘*comida*’ (i.e., food) along with the adjunct-PP ‘*para los cabritillos*’ (i.e., for the young goats).

**Table:** 

(8) (Manuela)	va	a hacer otra a mamá
(Manuela)	go.3p.sg.pres	to do another to mummy
‘(Manuela) is going to do another one for mummy’		
[Manuela, 2;02, the Deuchar corpus, CHILDES]		

**Table:** 

(9) Va	a	comprar mucha comida	para	los	cabritillos
go.2p.sg.pres	to	buy.inf. much food	for	the	young goat.pl
‘He is going to buy a lot of food for the young goats’					
[Leo, 3;00, the FerFuLice corpus, CHILDES]					

In dative CD structures (10), the verb ‘*voy a coger*’ (i.e., I am going to take) subcategorizes for the DO-DP ‘*los juguetes*’ (i.e., the toys) and the IO-PP ‘*Paloma*’ followed by the preposition ‘*a*’ (i.e., to). This prepositional complement is co-indexed with the dative clitic ‘*le*’ (i.e., her) in person, number, theta role and Case properties. We follow Demonte (1995) in accounting for the overt or the null realization of dative clitics in third person (singular or plural) for the formation of Spanish dative CD and NCD, respectively. In other words, this alternation only occurs with third person dative clitic clitics since ‘in first and second person dative constructions, the clitic is unavoidable given that clitics are obligatory when the “double” is a pronoun: *Te entregaron (a ti) a tus enemigos* (cf. they hand you over to your enemies)’ (Demonte, 1995, p. 6).

**Table:** 

(10) **Le**	voy	a coger	los juguetes a Paloma
her.cl.dat	go.1p.sg.pres	a take	the toys to Paloma
‘I am going to take the toys from Paloma’			
[Irene, 2;02, the LlinasOjea corpus, CHILDES]			

We have also considered dative CD constructions for which dative clitics in the third person combine with pronominal accusative clitics and, via a dissimilation process, these dative clitics adopt an impersonal dative ‘*se*’ form (Aranovich, 2012). This is illustrated in (11), that is, the dative clitic *‘le*’ has combined with the masculine accusative clitic ‘*lo*’ (i.e., it) and, as a result of dissimilation, the dative clitic is phonologically realized as the impersonal dative pronoun ‘*se*’. These constructions have been especially observed in the child-directed speech.

**Table:** 

(11) Se	lo	das	(a Leo)
him.cl.dat	it.cl.acc	give.2p.sg.pres	to Leo
‘You give Leo it’			
[Ivo, the FerFuLice corpus, CHILDES]			

The classification of the two constructions under analysis has been executed irrespective of the linear distribution and the DP or the complementizer phrase forms of the two internal constituents and adjuncts. Two chief measures have been used in this study to explore the two RQs formulated in Sect. 5.1. On the one hand, in order to examine the emergence of Spanish dative CD and NCD in the three child groups, the age of first occurrence has been considered following Snyder and Stromswold’s (1997, p. 287) premises: ‘age of first use is highly correlated with other measures of acquisition and it is the most sensitive measure of grammatical competence available from production data’. On the other hand, Mean Length of Utterance (MLU) has been used as a measure to explore the developmental incidence of the two target constructions in the children’s output. Developmental production patterns were analyzed taking into consideration 4 stages based on MLU values measured in words (MLUw, henceforth) (Brown, 1973). These developmental stages have taken as a point of departure Brown’s (1973) stages and have been analyzed by MLU using the CLAN (Computerized Language Analysis) software hosted in CHILDES. This program enables measuring the children’s MLU for each of the files available in the corpus under analysis in the three child groups. The MLUw stages range from 1 to 4 words. Given the fluctuations concerning the MLUw values reflected in each child’s output, as observed in Table 3 in the files analyzed for Manuela in the production of NCD structures, the MLUw onset stage has been determined when the children’s MLUw values display each of the 4 MLUw stages established for this study period, namely, MLUw 1, MLUw 2, MLUw 3 and MLUw 4. Based on these MLUw stages, we have analyzed the longitudinal production of Spanish dative CD and NCD per MLUw stage with a view to examine the incidence of these constructions through language development.

**Table 3:** Sample of the MLUw calculation for MLUw stage 2 per file based on Manuela’s production of non-clitic doubling structures (the Deuchar corpus)

Utterance	File MLUw	File name
*CHI: no tenemos otro collar para Granny	2.111	870829 sp.cha
*CHI: M va a hacer otra a mamá	2.111	870829 sp.cha
–	2.498	871129 sf.cha
–	2.034	871226 sf.cha

In our study, we have excluded a few instances that do not follow our data selection requirements discussed in Sect. 5.1.2 and, in particular, those ones concerned with the declarative canonical order that underlies the two internal constituents and adjuncts in dative CD and NCD.^13^ For example, repetitions (that is, when the child repeats verbatim what the adult just said) and locative monotransitive utterances (12) since the PP denotes location instead of a goal or a beneficiary theta role, as it is the case of prepositional dative NCD with ‘*a*’ and ‘*para*’, respectively.

**Table:** 

(12) Lo	hemos	tirado	a	la basura
it.acc.cl	have.1p.pl.pres	thrown.part	to	the rubbish
‘We threw it in the rubbish’				
[Emilio, 3;01, the Vila corpus, CHILDES]				

### Results

The children’s production of the two Spanish dative NCD and CD constructions represents low frequency rates out of the total verbal and non-verbal utterances produced by the HL bilinguals, the L1 bilinguals and the Spanish monolinguals in the files analyzed for this study. This pattern is also seen in the child-directed speech of the three child groups, as depicted in Table 4 for each child.

**Table 4:** Production of Spanish dative (non-)clitic doubling predicates per child and child-directed speech out of the (non-)verbal utterances examined in the files analyzed (# of cases (%))

Child group		Dative NCD	Dative CD	Total (non-)verbal utterances	# files analyzed in CHILDES
HL bilinguals	Manuela Manuela’s child-directed speech	2 (0.14%) 9 (0.32%)	5 (0.35%) 160 (5.72%)	1,435 (100%) 2,798 (100%)	9 (all the files available in CHILDES)
	Carla Carla’s child-directed speech	4 (0.44%) 1 (0.09%)	8 (0.88%) 67 (5.87%)	905 (100%) 1,142 (100%)	21 (all the files available in CHILDES)
L1 bilinguals	Leo Leo’s child-directed speech	2 (0.04%) 85 (1.58%)	11 (0.24%) 743 (13.82%)	4,497 (100%) 5,376 (100%)	32 (out of 61 CHILDES files)
	Simon Simon’s child-directed speech	1 (0.03%) 85 (1.58%)	4 (0.11%) 743 (13.82%)	3,650 (100%) 5,376 (100%)	32 (out of 61 CHILDES files)
Spanish monolinguals	Irene Irene’s child-directed speech	43 (0.36%) 103 (0.63%)	297 (2.49%) 1,196 (7.33%)	11,900 (100%) 16,314 (100%)	60 (all the files available in CHILDES)
	Emilio Emilio’s child-directed speech	2 (0.13%) 40 (2.02%)	93 (6.10%) 429 (21.68%)	1,524 (100%) 1,979 (100%)	11 (out of 25 CHILDES files)

However, when considering the amount of production of the two predicates under analysis overall (Table 5), substantial divergences are seen in the two bilingual child groups and also when the bilinguals are compared to the monolingual children. These results are reflected in the Spanish monolinguals’ greater production of dative CD and NCD predicates (435 cases), when compared to the use of the two target constructions in the spontaneous production of the HL bilingual children (19 cases) and the L1 bilingual children (18 cases). Differences also appear in the adults’ speech each child group interacts with, namely, the adults that interact with the Spanish monolinguals (1768 cases), the HL bilinguals (237 cases) and the L1 bilinguals (1656 cases). As a result of the differences in the amount of data available in CHILDES for the child-directed speech in the two bilingual child groups, the L1 bilingual children receive a higher adult exposure to the two target structures with respect to that observed in the HL bilingual children, a pattern that is not seen in the speech of the two bilingual child groups, as discussed earlier.

**Table 5:** Overall production of Spanish dative (non-)clitic doubling predicates (# of utterances (%))

	HL bilinguals				L1 bilinguals			Monolinguals		
	Dative NCD	Dative CD	Total dative (N)CD		Dative NCD	Dative CD	Total dative (N)CD	Dative NCD	Dative CD	Total dative (N)CD
Children	6 (32%)	13 (68%)		19 (100%)	3 (17%)	15 (83%)	18 (100%)	45 (10%)	390 (90%)	435 (100%)
Child-directed speech	10 (4%)	227 (96%)		237 (100%)	170 (10%)	1486 (90%)	1656 (100%)	143 (8%)	1625 (92%)	1768 (100%)

As shown in Table 5, an analogous distribution regarding the higher frequency rates in the overall production of dative CD predicates when compared to NCD predicates is equally observed in the child-directed speech and in the child output of the three groups under analysis. As for the input analysis, the monolingual children’s two parents, as well as the twins’ father living in Spain (that is, the L1 Spanish children) represent monolingual caregiver exposure to CD and NCD. In particular, NCD occurs in 8–10% of key utterances in child-directed speech, meaning that 90–92% contained CD in the input. These patterns are the monolingual baseline. In addition, the two HL bilingual girls’ input to mostly Spanish in the home (only Spanish by parents and English through babysitter and grandmother), however, indicates slightly more CD (96%) as only 4% of the key utterances in the input were NCD. Therefore, the production of Spanish CD and NCD in the child-directed speech does not exhibit a great difference between the three groups and NCD structures are only an exception given that most often parents expose children to CD predicates. Differences are not seen either in the three child groups regarding the production patterns of the target constructions (namely, CD > NCD). The Spanish monolingual children are the only group that seems to match their input by using CD in 92% of all key utterances. The L1 bilingual children showed lower production rates in CD (83% of all key utterances) and, finally, the HL bilinguals marked the least frequency rates of CD (68% of all key utterances). Thus, although the children of the three groups hear relatively frequent use of CD out of all the target constructions in their parental input in Spanish, they use less and less amount of CD utterances (relative to all the key utterances they produce) when comparing across child groups: monolinguals > L1 bilinguals > HL bilinguals.

With regards to the age of first clear use (Table 6), a sequential order in the onset of the two constructions is reflected in the two bilingual groups alike, except for Carla (an HL bilingual child) who shows a concurrent emergence of dative CD and NCD at 2;04. More specifically, dative CD utterances appear earlier than their dative NCD counterparts in the HL bilinguals and the L1 bilinguals, and a similar order effect in their onset is seen in the Spanish monolingual children.

**Table 6:** Age of first occurrence of Spanish dative (non-)clitic doubling structures

HL bilinguals			L1 bilinguals			Monolinguals		
Child	Dative CD	Dative NCD	Child	Dative CD	Dative NCD	Child	Dative CD	Dative NCD
Manuela	2;00	2;02	Leo	2;05	2;11	Emilio	1;09	2;08
Carla	2;04	2;04	Simon	2;01	2;11	Irene	1;09	2;02
Mean age	2;02	2;03	Mean age	2;03	2;11	Mean age	1;09	2;05

**Fig. 1:**
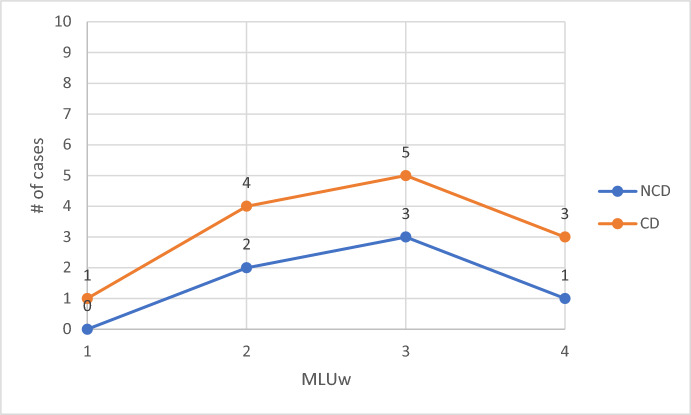
The HL bilinguals’ incidence of dative (non-)clitic doubling utterances per MLUw stage

**Fig. 2:**
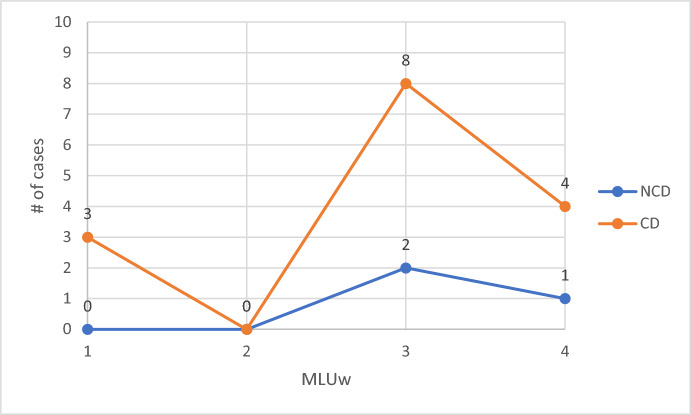
The L1 bilinguals’ incidence of dative (non-)clitic doubling utterances per MLUw stage

Although a sequential order occurs in the onset of dative CD and NCD, results show an emergence of the two predicates at around the age of 2 in the HL bilingual children and in the L1 bilingual children. This is evidenced by the absence of statistically significant differences in the ages of first occurrence of the two constructions in the two bilingual child groups (*z* = -1.000, *p* = 0.317 in the HL bilinguals; *z* = -1.342, *p* = 0.180 in the L1 bilinguals; Wilcoxon signed rank test). This age of emergence is in line with the data analyzed in their Spanish monolingual counterparts (*z* = -1.342, *p* = 0.180; Wilcoxon signed rank test). Such an order effect in the onset is also seen when the incidence of these structures is analyzed through language development, as measured by MLUw stages. As depicted in Fig. 1 (the HL bilinguals) and in Fig. 2 (the L1 bilinguals), higher production rates are observed in dative CD with respect to dative NCD in the two bilingual child groups.

**Fig. 3:**
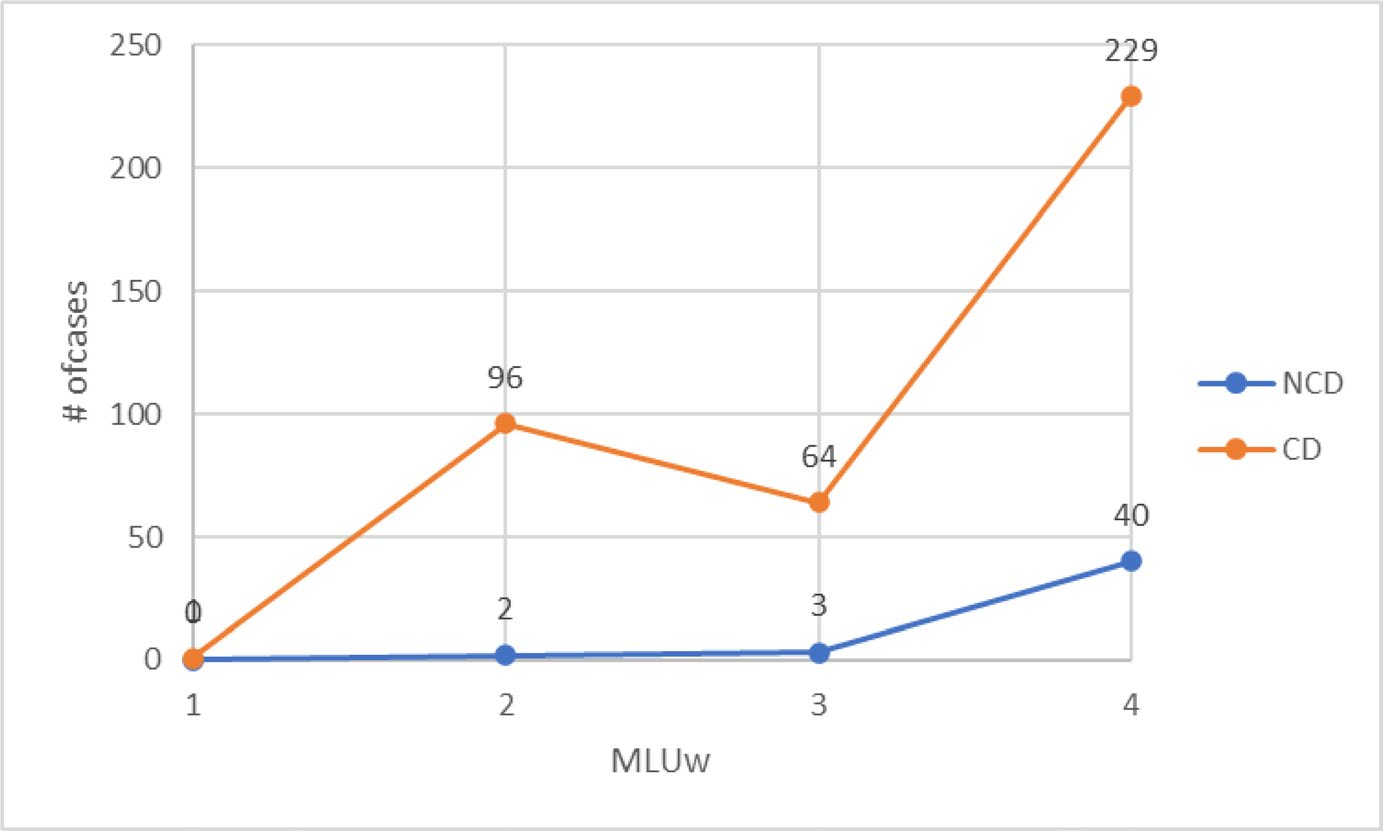
The Spanish monolinguals’ incidence of dative (non-)clitic doubling utterances per MLUw stage

The Spanish monolingual children exhibit a similar developmental incidence pattern, as in Fig. 3. In particular, dative CD constructions are more frequently produced from the stage at which these children reach an MLU of 1 word to the stage at which they reach an MLU of 4 words. On the contrary, a low incidence is reflected from the MLUw of 1 that gradually increases until an MLU of 4 words is reached.

**Fig. 4:**
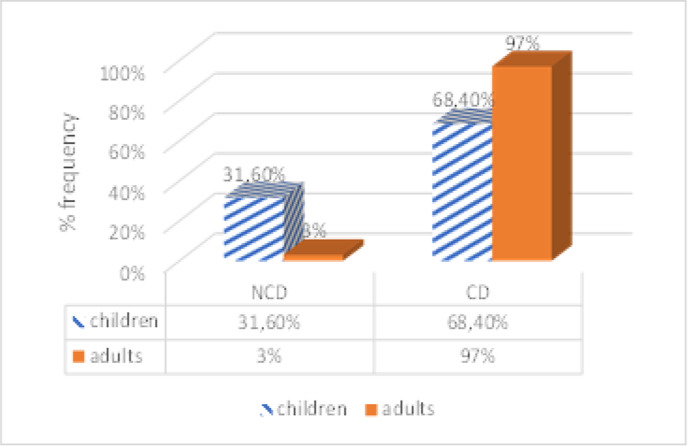
The HL bilingual children’s output and child-directed speech [100% = overall production of dative (non-)clitic doubling utterances in the child output and the child-directed speech]

**Fig. 5:**
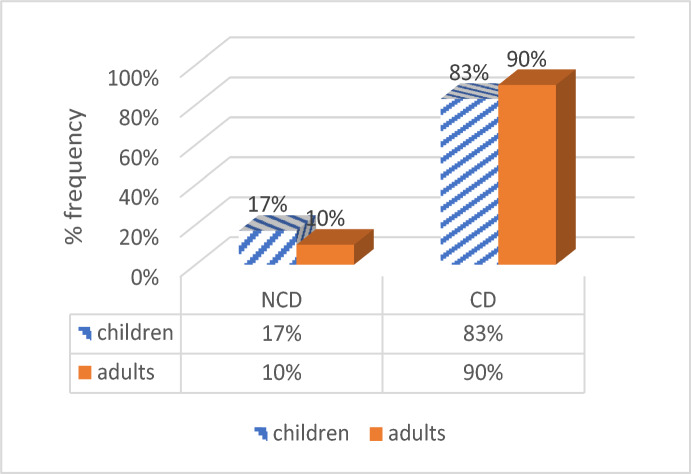
The L1 bilingual children’s output and child-directed speech [100% = overall production of dative (non-)clitic doubling utterances in the child output and the child-directed speech]

**Fig. 6:**
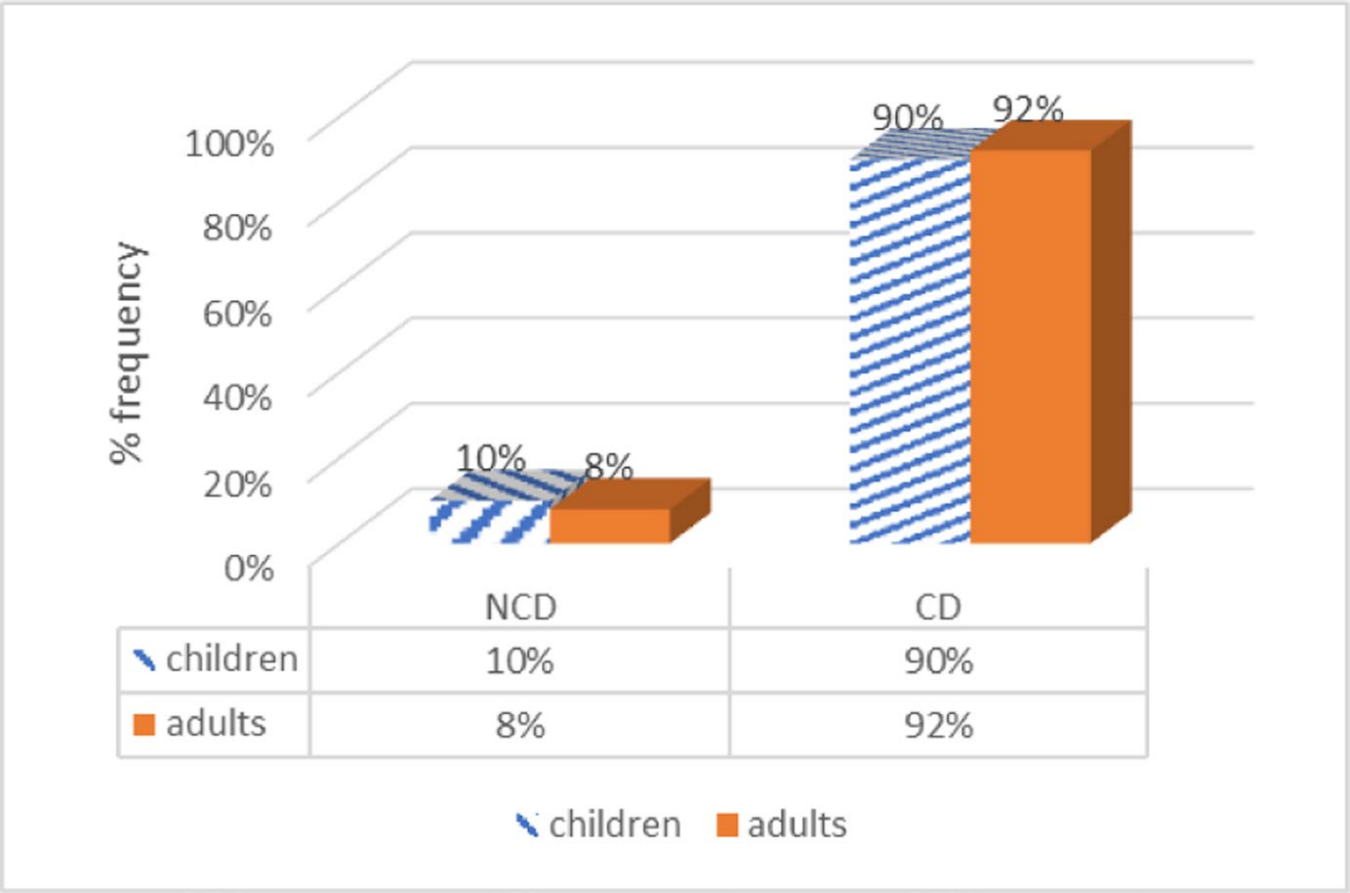
The Spanish monolingual children’s output and child-directed speech [100% = overall production of dative (non-)clitic doubling utterances in the child output and the child-directed speech]

The developmental production pattern observed in the bilingual and the monolingual children’s use of dative CD and NCD goes hand in hand with the production patterns reflected in the child-directed speech of the three child groups. Adults prefer the production of dative CD constructions over dative NCD structures with ‘*a*’ and ‘*para*’. These findings are displayed in Fig. 4 for the HL bilinguals (227 dative CD (97%) > 10 dative NCD with ‘*a*’ and ‘*para*’ (3%) in the child-directed speech; 13 dative CD (68.4%) > 6 dative NCD with ‘*a*’ and ‘*para*’ (31.6%) in the child output), in Fig. 5 for the L1 bilinguals (1486 CD (90%) > 170 NCD with ‘*a*’ and ‘*para*’ (10%) in the child-directed speech; 15 dative CD (83%) > 3 dative NCD with ‘*a*’ and ‘*para*’ (17%) in the child output) and in Fig. 6 for the Spanish monolinguals (1625 dative CD (92%) > 143 dative NCD with ‘*a*’ and ‘*para*’ (8%) in the child-directed speech; 390 dative CD (90%) > 45 dative NCD with ‘*a*’ and ‘*para*’ (10%) in the child output).

Table 7 displays the children’s output of dative CD and NCD utterances in the three child groups as well as the production of these predicates in their respective child-directed speech.

**Table 7:** The production of Spanish dative (non-)clitic doubling structures in the output of each bilingual and monolingual child and in the child-directed speech (# of cases (%))

Language group	Children		Children’s output of dative CD	Child-directed speech of dative CD	Children’s output of dative NCD with ‘*a*’ and ‘*para’*	Child-directed speech of dative NCD with ‘*a*’ and ‘*para’*
HL bilinguals	Carla		8 (66.7)	67 (98.5)	4 (33.3)	1 (1.5)
	Manuela		5 (71.4)	160 (94.7)	2 (28.6)	9 (5.3)
Subtotal (overall)			13 (68.4)	227 (97)	6 (31.6)	10 (3)
L1 bilinguals	Leo		11 (84.6)	743 (89.8)	2 (15.4)	85 (10.3)
	Simon		4 (80)	743 (89.8)	1 (20)	85 (10.3)
Subtotal (overall)			15 (83)	1486 (90)	3 (17)	170 (10)
Spanish monolinguals		Emilio	93 (97.9)	429 (91.5)	2 (2.1)	40 (8.5)
		Irene	297 (87.4)	1196 (92)	43 (12.6)	103 (8)
Subtotal (overall)			390 (90)	1625 (92)	43 (10)	143 (8)

Thus, the greater frequency rates in the use of dative CD over NCD predicates, as reflected in the data illustrated in Figs. 1, 2, 3 (overall) and in Table 5 (per child), provide further insights into the correlation that occurs between the child output and their corresponding child-directed speech in the two bilingual groups as well as in the Spanish monolinguals. However, although the HL and the L1 bilinguals exhibit a similar pattern, dative CD utterances being more commonly produced, the HL bilinguals produce more dative NCD utterances with ‘*a*’ and ‘*para*’ although not significantly so (vs. L1 bilinguals, *U* = 1.55, *p* = .167; vs. the adult input they are exposed to, *U* = 1.34, *p* = .250 Mann–Whitney test; see Table 6). These potential differences between the two bilingual child groups are preliminary but worthy of future consideration in that Spanish is both an HL and an L1 for the HL group.

### Discussion of Results

In order to shed light on RQ 1, our findings have evidenced that the difference in the input quantity and quality that the HL bilingual children and the L1 bilingual children have been exposed to has not been an interfering factor in the occurrence of dative CD and NCD constructions at the age of 2. These emergence patterns are observed in the two bilingual child groups and in their Spanish monolingual peers. The results obtained in our study are in line with the findings accounted for by previous empirical works that have analyzed the emergence of Spanish dative CD and NCD structures in the Spanish–English bilingual children overall (that is, their focus of study has not been the family input versus the community input) and the Spanish monolinguals (e.g., Fernández Fuertes & Sánchez Calderón, 2021), as has been analyzed in the spontaneous production of dative clitic and non-clitic doubling predicates available in CHILDES. The fact that the exposure to Spanish has been rooted in a double input source (that is, at home and as the language of the community) in the L1 bilinguals or exclusively at home in the HL bilinguals has not triggered differences between the two bilingual child groups in the acquisition process of the grammatical underpinnings that relate dative CD and NCD constructions with ‘*a*’ or ‘*para*’. Hence, the family and/or the community input that the HL and the L1 bilingual children are exposed to in Spanish from early on does not seem to have caused differences between the two bilingual child groups in acquiring the common underlying structure upon which Spanish dative CD and NCD predicates are construed. As also reported by Fernández Fuertes and Sánchez Calderón (2021), these results comply with the so-called Complex Predicate Parameter proposed by Snyder (2001) and are attested by the ages of first clear occurrence of these two structures at around 2;00. More specifically, an underlying SC or complex predicate has been projected in the formation of dative CD and NCD constructions in the two bilingual groups and in the monolingual group alike. Although the two constructions at stake begin to be produced at around a similar age, an order effect has been evidenced in the children’s later occurrence and lower production of dative NCD utterances through the MLUw stages established, when compared to dative CD. These outcomes have been equally seen in the HL bilinguals, in the L1 bilinguals and in the Spanish monolinguals, as also attested by previous works (e.g., Torrens & Wexler, 2000). Thus, the data observed in the three child groups are in line with the acquisition of the syntactic underpinnings of dative NCD constructions and, in particular, the mediated role of ‘*a*’ and ‘*para*’ in allocating Case and theta role to the prepositional complement by the verbal head, as also reported by Sánchez Calderón and Fernández Fuertes (2020) in Spanish–English bilingual children overall and in Spanish monolingual children. Hence, the findings derived from RQ 1 suggest that the status of Spanish as an HL does not make these children follow a different path from that of L1 bilinguals, both behaving as their monolingual counterparts.

With regards to RQ 2, the HL and the L1 bilinguals’ child-directed speech has had a positive effect on the children’s developmental incidence and overall production of both constructions. That is, child output is in tune with the greater use of dative CD utterances and the lower use of their alternating prepositional dative NCD counterparts. This is the case regardless of whether Spanish has been exposed to only at home, as in the HL bilingual children, or via a double input source, namely, in the home context and as the language of the community, as in the L1 bilinguals. Analogous child-directed speech-child output production patterns of dative CD and NCD are also mirrored in their corresponding Spanish monolingual peers. Moreover, the low exposure to prepositional dative NCD utterances in the child-directed speech can also account for the later ages of first occurrence of these predicates in the three target child groups. Therefore, these results point to the fact that the input that the HL bilinguals, the L1 bilinguals and the Spanish monolinguals receive at home is optimal and has equally contributed to the emergence order effect of the two target constructions, as also reported by a great bulk of studies (e.g., Fernández Fuertes and Liceras, 2018; Sánchez Calderón & Fernandez Fuertes, 2020; Yang, 2016). The monolingual-like emergence findings that have been equally observed in the two bilingual child groups have helped to tease apart the debate arisen in the literature regarding the acquisition paths of Spanish as an HL or as an L1. This means that, despite the fact that the HL has been traditionally termed as the weaker or non-dominant language from the viewpoint of linguistic proficiency (e.g., Pires & Rothman, 2009), the HL bilinguals have not differed from the L1 bilinguals, and their corresponding Spanish monolingual peers, in the acquisition of dative CD and NCD in Spanish. This could indicate that the input quantity and quality (that is, how complex it is in terms of morphosyntax or lexicon) that the HL bilinguals have received from early on have not interfered in the production of both structures, as similar results have been observed in the adult input-child output patters in the two bilingual child groups and the Spanish monolingual children. One of the factors that can account for the HL bilingual children’s similar findings with respect to their Spanish monolingual counterparts is the exposure to this language from birth and in a naturalistic context (Montrul et al., 2015). This means that age of acquisition and early language exposure are two crucial factors in HL bilingualism and L1 bilingualism. These results are not only evidenced in the syntactic field, as it is the case of this study, but also in the morphosyntax domain (Montrul et al., 2015).

## Conclusions

The results obtained have been compared to Spanish monolingual children. As evidenced by the data analyzed in the two bilingual groups, dative CD and NCD utterances emerge at around the age of 2, as it is also the case in the Spanish monolingual children. This hints that the dual input source in Spanish (i.e., in the home setting and as the community language) in the L1 bilinguals or the reduced input source (i.e., exclusively at home) in the HL bilinguals have not been an interfering factor for acquiring the syntactic underpinnings of dative (non-)clitic doubling constructions in Spanish: (a) the overt or the null realization of the dative clitic ‘*le*’ or ‘*les*’ in the projection of dative CD and NCD, respectively, as evidenced by the adult-like utterances in the children’s speech; and (b) the syntactic relational status of the two constructions at stake, namely, the formation of a common underlying SC or complex predicate upon which both structures are construed, as attested by the absence of statistically significant differences in the emergence of the two structures. In addition to these grammatical properties, the two bilingual child groups also reflect monolingual-like acquisition patterns in the delayed emergence of the syntactic properties that underlie dative NCD and, in particular, the special Case and theta role mediated assignment status of the prepositions ‘*a*’ and ‘*para*’. This is evidenced by the later ages of first occurrence and the lower frequency rates in the production of dative NCD in contrast to dative CD. The child-directed speech appears to be in line with the two bilingual child groups’ incidence patterns through language development and overall with respect to the production of the two Spanish dative CD and NCD predicates. This is reflected in the relative frequency order, namely, the higher use of dative CD when compared to dative NCD. What is more, the adults’ preference order in the production of the two target structures also goes hand in hand with the order observed in the children’s emergence of these constructions, as equally attested in the three child groups. Therefore, these findings provide further insights into child-directed speech effects on the exposure to syntactic constructions and the order of appearance of (non-)clitic doubling structures in the children’s speech. Our results have also contributed to elucidate the debate concerning the monolingual-like acquisition patterns (e.g., Pires & Rothman, 2009) or the divergences (e.g., Toribio, 2004) that the HL bilingual children and the L1 bilingual children present with regards to the emergence and the incidence of dative CD and NCD. Thus, in the case of the HL bilingual children, Spanish being acquired as an HL from birth in the home context does not seem to have reflected a weak linguistic competence, as has been traditionally reported by earlier studies (e.g., Hurtado & Montrul, 2021; Montrul, 2016). Despite the reduced input that the HL bilingual children have received in this language when compared to the L1 bilingual children, the monolingual-like acquisition patterns that these children exhibit have been aided by the exposure to Spanish (and English, their other L1) in a naturalistic setting (e.g., Montrul et al., 2015). Further research could examine whether the acquisition of dative (non-)clitic doubling constructions in the bilingual children’s other L1 (that is, English) is determined by family and/or community input factors. The results obtained in the two bilingual child groups will also be compared to English monolingual children. These findings will allow to carry out an interlinguistic analysis in which directionality of crosslinguistic effects in the bilinguals’ two languages can be investigated. Given the type of data analyzed in our study, in particular, and the data available in the CHILDES project, in general, a broader selection of children and their corresponding adult input will help broaden the picture of the developmental patterns examined in the two bilingual groups and in the monolingual group. In addition, further research will explore whether the longitudinal incidence of the constructions under scrutiny at each MLUw stage is comparable among stages, between child groups and their corresponding child-directed speech. Further research will also shed light on whether differences (or lack thereof) between the two bilingual child groups and the monolingual group are exhibited in the acquisition of each alternating construction pair based on their verbal subcategorization framework, that is, a ditransitive verb in dative CD constructions that undergo alternation as dative NCD with ‘*a*’ in contrast to a monotransitive verb in dative CD predicates that undergo alternation as dative NCD with ‘*para*’ (Cuervo, 2003).

## Data Availability

All the data analyzed in this study are freely available online in the CHIldes Language Data Exchange System (MacWhinney, 2000): https://childes.talkbank.org/. In particular, we have analyzed data from three English–Spanish bilingual corpora, namely, the Deuchar corpus (https://childes.talkbank.org/access/Biling/Deuchar.html), the FerFuLice corpus (https://childes.talkbank.org/access/Biling/FerFuLice.html) and the Perez corpus (https://childes.talkbank.org/access/Biling/Perez.html). Likewise, we have also extracted data from two Spanish monolingual corpora, namely, from the LlinasOjea corpus (https://phon.talkbank.org/access/Spanish/LlinasOjea.html) and from the Vila corpus (https://childes.talkbank.org/access/Spanish-nomor/Vila.html).

## Footnotes

1 In this study, the term ‘community input’ refers to the language exposure that the HL bilingual children, the L1 bilingual children and the monolingual children receive in Spanish outside of the home context (e.g., through crèche). Further information about the source of community input the three child groups are exposed to is detailed in Table 1 (Sect. 5.2).

2 When the IO is a pronoun, the optional overt or null realization of the dative clitic cannot occur in Spanish (N)CD constructions. Hence, in utterances such as ‘*Le di un libro a él*’ (cf. I gave him a book), the dative clitic ‘*le*’ is always obligatory. These utterances have not been examined in this study since the focus of this work is the acquisition of constructions that undergo dative clitic alternation.

3 The Complex Predicate Parameter (Snyder, 2001), like any parameter, divides languages into two typological groups: [+ complex predicate] languages and [- complex predicate] languages. English is analyzed as a [+ complex predicate] language and, in the specific case of the structures under consideration here (i.e., Spanish (non-)clitic doubling structures), it is so because it has double object constructions. Spanish, on the other hand, is a [- complex predicate] language because it lacks English-like double object constructions.

4 The present study has set the basis on Sánchez Calderón and Fernández Fuertes (2020). However, their study has not distinguished input differences in the children’s selection criteria, that is, whether Spanish has been exposed to in the family context or as the societal language. Instead, Sánchez Calderón and Fernández Fuertes (2020) analyze the 9 Spanish–English bilingual children overall and also compare the data obtained to a selection of 9 Spanish monolingual children. Further details about the data selection criteria followed in the present study are discussed in Sect. 5.1.1.

5 The special status of the prepositions ‘*a*’ and ‘*para*’ has been reported for English to/for-datives (Snyder & Stromswold, 1997). This has been statistically evidenced by the correlation between English monolingual children’s concurrent emergence of to*-*datives and other related dative constructions such as dyadic to-datives at 2;06 (*r* = .83, *p* = .0009).

6 We follow the CHILDES format (MacWhinney, 2000) in referring to the children’s years and months.

7 Following Montrul (2016), optimal input is characterized as the adequate language balance that learners receive in terms of the following factors: (a) linguistic complexity: exposure to complex linguistic constructions in meaningful settings to encourage and challenge learners in language development; (b) comprehensibility: exposure to messages that learners can understand with the aid of contextual cues; (c) contextual relevance: exposure to meaningful contexts that relate to learners’ experiences and cultural identities; and (d) sufficient quantity and quality exposure to language: exposure to varied syntactic constructions, lexicon and communicative functions over time.

8 Meaningful and sufficient input are two relevant notions in HL and L1 acquisition. These notions focus on the relevance of offering learners with comprehensible and contextually relevant language input (that is, meaningful input) and in appropriate quantities (namely, sufficient input) (Montrul, 2010, 2016). With regards to the latter, despite the fact that there is no threshold for the amounts of language input to consider it as sufficient, previous longitudinal works (e.g., Hartshorn, Norman, Evans, Merrill, Sudweeks, Strong-Krause & Anderson, 2010) have evidenced that rich, varied and consistent language exposure facilitates language acquisition.

9 In order to carry out an age-based comparable selection of the two Spanish–English bilingual child groups, we have discarded the following corpora considering the data available in CHILDES to date. As for the HL bilingual children, we have discarded those children who produce only one dative (non-)clitic doubling predicate in Spanish, that is, either dative CD or NCD, as in the case of Alberto, John and Antonio (the Perez corpus), or who do not produce any of the two constructions under analysis, as in Sheila (the Perez Corpus) and Diego (the Ticio corpus). Furthermore, we have excluded the LlinasOjea corpus and, in particular, the data from two children, namely, Jordina and Yasmin, since they have received exposure to three languages from birth: Spanish, English and Catalan. We have also discarded the Reyes/Ervin-Tripp corpus and the SilvaCorvalan corpus given that, although they meet the criterion of Spanish and English exposure from early on, they contain data from an older age range than the one selected for the present study. That is, the Reyes/Ervin-Tripp corpus involves child data from 7;00 and 10;00 year-olds and the children’s ages in the SilvaCorvalan corpus range from 5;00 to 6;00. In the L1 bilingual children, the set of Spanish–English bilingual twins produce both dative CD and NCD predicates along the study period (1;01–3;01). There are no other corpora available in CHILDES that include data from children who meet the criteria to be part of this bilingual group.

10 In the CHILDES metadata, although Manuela’s mother’s L2 Spanish proficiency level is not specified, the utterances analyzed in this study do not reveal the grammatical properties of an L2 speaker.

11 Although the metadata in CHILDES dot not specify whether Irene and Emilio attended crèche and whether the parents’ Spanish was the L1 or the L2, both children were born and raised in a monolingual Spanish-speaking environment at home and in the community.

12 In our Spanish monolingual corpora selection, we have discarded (a) longitudinal corpora that involved child data from an older age range (e.g., the Col/Mex corpus (age 6;00–7;00), the Díez-Itza corpus (age 3,00–3;11), the Fernández/Aguado corpus (age 3;00–4;00), the GRERLI corpus (age 9;00–18;00), the Hess corpus (age 6;00–12;00) and the Shiro corpus (age 6;00–9;00); (b) cross-sectional corpora (e.g., the BeCaCeSno corpus (age intervals 3;06–11;06), the Jackson/Thal corpus (age intervals 0;10–3;00) and the Romero corpus (age interval 2;00); (c) those corpora that involve child data with a short age range that will not allow us to analyze the child linguistic development: Juan (1;10–2;07; the OreaPine corpus), Koki (1;07–2;11; the Montes corpus), Lucía (2;02–2;07; the OreaPine corpus) and Mendía (1;08–2;02; the Nieva corpus); (d) those corpora that do not offer data of the early stages of language acquisition: Juan (2;00–4;00; the Linaza corpus) and Idaira (2;07–4;07; the Marrero corpus); and (e) María (the Ornat corpus) which, although she meets the age range selection requirements for our study, we will exclude her data to achieve comparability in terms of the amount of children analyzed across the three age groups (N = 2).

13 As a result of the variable constituent order in Spanish, we refer to declarative canonical order to those dative CD and NCD utterances in which the internal constituents and adjuncts present the following combinations: (a) a DO-DP followed by an IO-PP (e.g., ‘*Mark ****le**** enseñó una foto memorable a su hijo*’; cf. Mark showed his son a memorable picture) in dative CD or ‘*Mark enseñó una foto memorable a su hijo*’ (cf. Mark showed a memorable picture to his son) or an IO-PP along with a DO-DP (e.g., ‘*Mark ****le**** enseñó a su hijo una foto memorable*’; cf. Mark showed his son a memorable picture) in dative CD or ‘*Mark enseñó a su hijo una foto memorable*’ (cf. Mark showed a memorable picture to his son) in dative NCD with ‘*a*’; or (b) a DO-DP followed by an adjunct-PP (e.g., ‘*Mark ****le**** preparó la merienda a su hijo*’; cf. Mark made his son a snack)) in dative CD or ‘*Mark preparó la merienda a su hijo*’ (cf. Mark made a snack for his son) in dative NCD with ‘*para*’ or an adjunct-PP and a DO-DP (e.g., ‘*Mark ****le**** preparó a su hijo la merienda*’; cf. Mark made his son a snack) in dative CD or ‘*Mark preparó a su hijo la merienda*’; cf. Mark made a snack for his son) in dative NCD with ‘*para*’.
